# The Role of Minimally Invasive Surgery in Gastric Cancer

**DOI:** 10.7759/cureus.19563

**Published:** 2021-11-14

**Authors:** Nail Omarov, Derya Uymaz, Ibrahim F Azamat, Emre Ozoran, Ibrahim H Ozata, Fatih S Bırıcık, Orhun C Taskin, Emre Balik

**Affiliations:** 1 General Surgery, Koç University Hospital, İstanbul, TUR; 2 General Surgery, Koç University Hospital, Istanbul, TUR; 3 Medical Oncology, Koç University Hospital, Istanbul, TUR; 4 Pathology, Koç University Hospital, Istanbul, TUR; 5 General and Colorectal Surgery, Koç University Hospital, Istanbul, TUR

**Keywords:** gastric cancer surgery, minimally invasive laparoscopy, robotic assited surgery, gastrectomy, oncology

## Abstract

Objective: This study aims to investigate the efficacy and safety of minimally invasive surgery (MIS) in gastric cancer and to compare MIS versus open gastrectomy (OG) in terms of early mortality and morbidity, long-term oncological outcomes, and recurrence rates.

Methods: A total of 75 patients who underwent MIS or OG for gastric cancer at Koç University School of Medicine between December 2014 and December 2019 were retrospectively analyzed. Postoperative complications and disease-specific survival were compared between surgical approaches.

Results: Of the patients, 44 were treated with MIS and 31 with OG. In the MIS group, 33 patients underwent laparoscopic surgery, and 11 patients underwent robotic gastrectomy. Duration of operation was significantly longer in the MIS group than in the OG group (p<0.0001). The median amount of blood loss was 142.5 (range, 110 to 180) mL in the MIS group and 180.4 (range, 145 to 230) mL in the OG group (p<0.706). The median number of lymph node dissection was 38.9 (range, 15 to 66) and 38.7 (range, 12 to 70) in the MIS and OG groups, respectively (p<0.736). The median length of hospitalization, twelve days in the OG group and nine days in the MIS group. Median follow-up was 19.1 (range, 2 to 61) months in the MIS group and 22.1 (range, 2 to 58) months in the OG group. The median OS and DFS rates were 56.8 months and 39.6 months in the MIS group, respectively (log-rank; p=0.004) and 31.6 months and 23.1 months in the OG group, respectively (log-rank; p=0.003).

Conclusion: Our study results suggest that, despite its technical challenges, MIS is an effective and safe method in treating gastric cancer with favorable early mortality and morbidity rates and long-term oncological outcomes, and acceptable recurrence rates.

## Introduction

According to the 2018 World Health Organization (WHO) data, gastric cancer is the fourth most common cancer in all age groups and both sexes [1]. It is the third leading cause of cancer mortality worldwide [1]. Despite recent developments in medicine, the likelihood of developing gastric cancer increases with advanced age, as the life expectancy has increased in most countries [2,3].

Open gastrectomy (OG) has been used as the standard surgical technique for many years in treating gastric cancer. Over the past three decades, however, minimally invasive surgery (MIS) has gained popularity. Laparoscopic gastrectomy was first introduced in 1994 [4]. For the last decade, the use of robotic surgery has become widespread worldwide thanks to its favorable early and late outcomes [5]. The main advantages of MIS include less early postoperative pain, shorter length of hospital stay, and improved long-term quality of life [6]. It has been shown that MIS is an effective and safe treatment method for early gastric cancer [6]. Although data are limited for advanced stages and no established indication, it can be successfully performed with favorable early oncological outcomes [7-10].

Previous studies comparing laparoscopic gastrectomy and OG have demonstrated that laparoscopic surgery yields less intraoperative bleeding, less postoperative pain, acceptable D2 lymph node dissection, shorter length of hospitalization, and similar survival rates as compared to OG [11,12]. Although the rate of minor complications such as wound infection, ileus, vomiting is lower, the major complication rate is like OG [10]. Robotic gastrectomy is superior to laparoscopic gastrectomy with better intraabdominal visualization, less intraoperative bleeding, and similar morbidity rates [13]. However, prolonged surgery and higher cost are the main disadvantages of robotic gastrectomy [14].

In the present study, we aimed to investigate MIS's efficacy and safety in gastric cancer and compare MIS versus OG in terms of early mortality and morbidity, long-term oncological outcomes, and recurrence rates.

## Materials and methods

**Figure 1:**
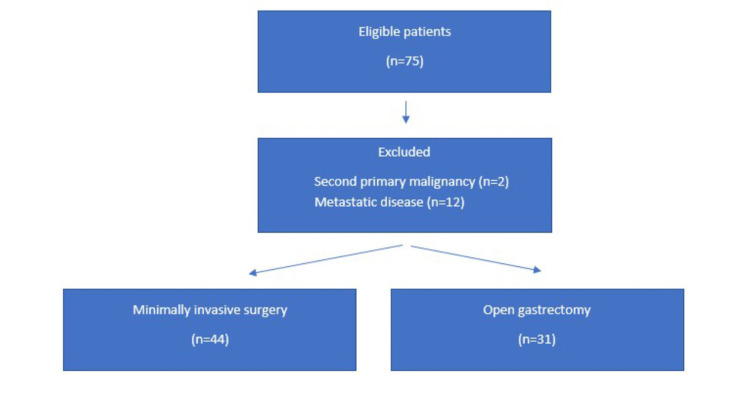
Study flow chart

This single-center, retrospective study was conducted at the Department of General Surgery of Koç University Hospital. Medical data of patients who underwent MIS or OG for gastric cancer in our center between December 2014 and December 2019 were retrospectively analyzed. Demographic data, surgical data, early surgical outcomes, pathological data, and survival data were retrospectively retrieved from the prospective database. The preoperative diagnosis was made based on endoscopic examination and gastric adenocarcinoma was confirmed histo-pathologically. Those patients with a second primary malignancy and metastatic disease were excluded. For staging, endoscopic ultrasound (EUS), computed abdominal, and chest tomography (CT) were routinely used; magnetic resonance imaging (MRI) and positron emission tomography (PET/CT) were selectively used. Finally, a total of 75 patients were included in the study. The study flow chart is shown in Figure 1.

Written informed consent was obtained from each patient. The study protocol was approved by the Koç University Ethics Committee No. 2021.058.IRB2.007. The study was conducted by the principles of the Declaration of Helsinki.

The patients diagnosed with ≥T2 gastric tumors as evidenced by imaging modalities or those with pathological lymph node positivity received neoadjuvant treatment. Based on the tumor location, total or distal subtotal gastrectomy and D2+ lymph node dissection were performed in accordance with the 2018 Japanese Gastric Cancer Treatment Guidelines [15]. A Roux-en-Y reconstruction was done for all reconstructions. Duration of operation, number of lymph node dissection, morbidity, mortality, length of intensive care unit (ICU) and hospital stay, major and minor complications, pathological results, overall survival (OS), and disease-free survival (DFS) rates were analyzed.

Treatment decisions were made based on the Multidisciplinary Tumor Board Consensus recommendations. During follow-up, the Clavien-Dindo classification system was used to evaluate postoperative complications [16]. All patients were scheduled for follow-up every three months for two years and assessed using contrast-enhanced abdominal and thoracic CT and using endoscopic examination annually.

Statistical analysis

Statistical analysis was performed using the SPSS for Windows version 25.0 software (IBM Corp., Armonk, NY, USA). Descriptive data were expressed in median (min-max) for continuous variables and in number and frequency for categorical variables. The Shapiro-Wilk test was used to assess the distribution of continuous data. For comparison of two normally distributed variables, the Student’s t-test was used. Comparisons between two non-normally distributed continuous variables were performed using the Mann-Whitney U test. The difference between more than two non-normally distributed continuous variables was analyzed using the Kruskal-Wallis test. The Kaplan-Meier analysis was carried out to analyze OS and DFS rates. A p-value of <0.05 was considered statistically significant.

## Results

Of a total of 75 patients, 44 were treated with MIS and 31 with OG. In the MIS group, 33 patients underwent laparoscopic surgery, and 11 patients underwent robotic gastrectomy. The baseline demographic and clinical characteristics of the patients are shown in Table 1.

**Table 1:** Baseline demographic and clinical characteristics of patients Data are given in median (min-max) or number and percentage unless otherwise stated. MIS: minimally invasive surgery; OG: open gastrectomy; BMI: body mass index; ASA: American Society of Anesthesiologists.

MIS (n=44)		OG (n=31)	
Variable	n, %	n, %	P-value
Age, years, median (range)	65.2 (37–90)	58.8 (29–78)	<0.276
Sex			<0.444
Male	29 (65.9%)	23 (74.1%)	
Female	15 (34.09%)	8 (25.8%)	
BMI, kg/m^2^, median (range)	24.34 (20–32)	26.18 (22–38)	<0.338
ASA class			
1	11 (25%)	10 (32.25%)	
2	20 (45.45%)	15 (48.38%)	
3	13 (29.54%)	6 (19.35%)	
4	0	0	

Thirty-seven total and seven subtotal gastrectomies were performed in the MİS group and 26 total gastrectomies were performed in the OG group. The median duration of operation was significantly longer in the MIS group, compared to the OG group (220.45 [range, 120 to 720] min vs. 171.06 [90 to 380] min, respectively; p<0.0001). The median amount of blood loss was 142.5 (range, 110 to 180) mL in the MIS group and 180.4 (range, 145 to 230) mL in the OG group (p<0.706). In the MIS group, 14 (31.81%) of the patients were converted to open gastrectomy due to dense adhesions related to previous surgery in 12 (27.7%) patients and due to poor visualization related to intraabdominal adiposity in two (4.54%) patients. In both groups, R0 resection was achieved. The median number of lymph node dissection was 38.9 (range, 15 to 66) and 38.7 (range, 12 to 70) in the MIS and OG groups, respectively (p<0.736). The pathological stages were classified according to the 8th Edition International Union Against Cancer (UICC) Tumor, Node, Metastasis (TNM) Classification of Malignant Tumors. Operative and intraoperative data are presented in Table 2.

**Table 2:** Operative and intraoperative data Data are given in median (min-max) or number and percentage unless otherwise stated. MIS: minimally invasive surgery; OG: open gastrectomy; TNM: tumor, node, metastasis. *Stage 0 included: 3 patient intramucosal tumor, 3 patients regressed than advanced stage tumor to stage 0 after neoadjuvant chemotherapy.

	MIS (n=44)	OG (n=31)	
Variable	n, %	n, %	P value
Tumor localization			
Cardia	9 (20.45%)	7 (22.58%)	
Corpus	14 (31.81%)	7 (22.58%)	
Antrum	20 (45.45%)	16 (51.6%)	
Linitis plastica	1 (2.27%)	1 (3.22%)	
Previous abdominal surgery	12 (27.27%)	12 (38.7%)	
Neoadjuvant treatment			
Yes	28 (63.64%)	16 (51.62%)	
No	16 (36.36%)	15 (48.38%)	
Type of surgery			
Total gastrectomy	37 (84.09%)	26 (83.8%)	
Subtotal gastrectomy	7 (15.9%)	5 (16.1%)	
Operation time, min, median (range)	220.45 (120–720)	171.06 (90–380)	<0.0001
T stage			
T0	6 (13.63%)	0	
T1	10 (22.72%)	5 (16.12%)	
T2	6 (13.63%)	3 (9.67%)	
T3	10 (22.72%)	3 (9.67%)	
T4	12 (27.27%)	19 (61.29%)	
Number of lymph node dissection, median (range)	38.9 (15–75)	38.7 (12–70)	<0.736
Amount of blood loss, mL, median (range)	142.5 (120–720)	180.4 (90–380)	<0.706
Switch to OG	14 (31.81%)	-	
TNM stage			
0	6* (13,6)	0	
ⅠA	9 (20.45%)	4 (12.9%)	
ⅠB	3 (6,81%)	3 (9.67%)	
ⅡA	9 (20.45%)	3 (9.67%)	
ⅡB	1 (2.27%)	1 (3.22%)	
ⅢA	5 (11.36%)	3 (9.67%)	
ⅢB	5 (11.36%)	3 (9.67%)	
ⅢC	6 (13.63%)	10 (32,25%)	
Ⅳ	0	4 (12,9%)	

Postoperative complications are summarized in Tables 3 and 4.

**Table 3:** Postoperative complications Data are given in median (min-max) or number and percentage unless otherwise stated. MIS: minimally invasive surgery; OG: open gastrectomy.

	MIS (n=44)	OG (n=31)	
Variable	n, %	n, %	P-value
Intraabdominal abscess	9 (20.4%)	12 (26.6%)	<0.047
Lung infection	6 (20%)	9 (20%)	<0.054
Anastomotic fistula	3 (6.81%)	2 (6.45%)	<0.453
Intraabdominal bleeding	3 (6.81%)	2 (6.45%)	
Pancreatic fistula	2 (4.54%)	1 (3.2%)	<0.133
Ileus	2 (6.66%)	5 (11.1%)	<0.453
Mortality	1 (2.27%)	1 (3.22%)	
Length of hospital stay, days, median (range)	9 (6–45)	12 (range 7–49)	<0.800

**Table 4:** The postoperative complications of Clavien-Dindo classification

Clavien-Dindo classification	MIS	OS
Grade 1	30 (%68.1)	18 (%58.06)
Grade 2	5 (%11.36)	4 (%12.9)
Grade 3	8 (%18.1)	6 (%19.35)
Grade 4	0	2 (%6.45)
Grade 5	1(%2.27)	2 (%6.45)

The median length of hospitalization was 12 days (8-49) in the OG group and 9 (6-45) days in the MIS group. The main causes of prolonged hospitalization were wound infection and poorly controlled postoperative pain in the OG group. Mortality was observed in two patients. One (2.27%) patient requiring ICU stay in the MIS group and another (3,22%) patient requiring ICU stay in the OG group died from pulmonary comorbidities and hematologic disorders, respectively.

**Figure 2:**
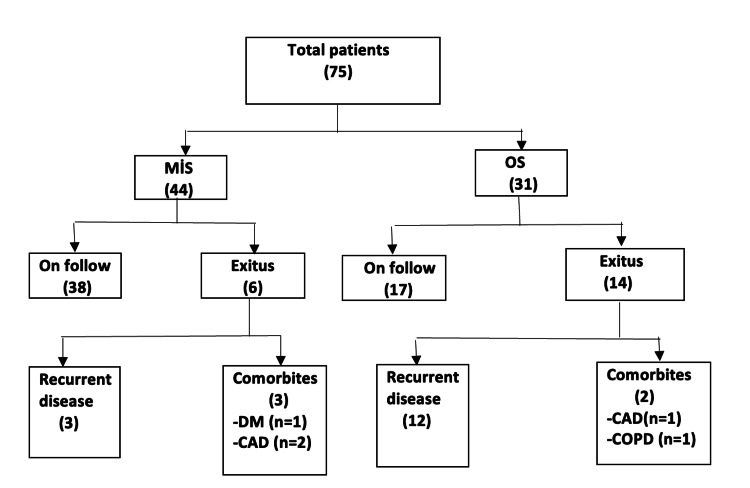
The long-term outcomes of the patients

The median follow-up was 19.1 (range, 2 to 61) months in the MIS group and 22.1 (range, 2 to 58) months in the OG group. Six (13.63%) of the patients treated with MIS died during follow-up. Three (6.81%) of them had recurrent disease, and the remaining three (6.81%) patients had comorbidities. In the OG group, 14 (45.16%) patients died during follow-up. Of these patients, 12 (38.7%) died due to systemic disease, while two (6.45%) had comorbidities in Figure 2.

The median OS and DFS rates were 56.8 months and 39.6 months in the MIS group, respectively (log-rank; p=0.004), and 31.6 months and 23.1 months in the OG group, respectively (log-rank; p=0.003).

**Figure 3:**
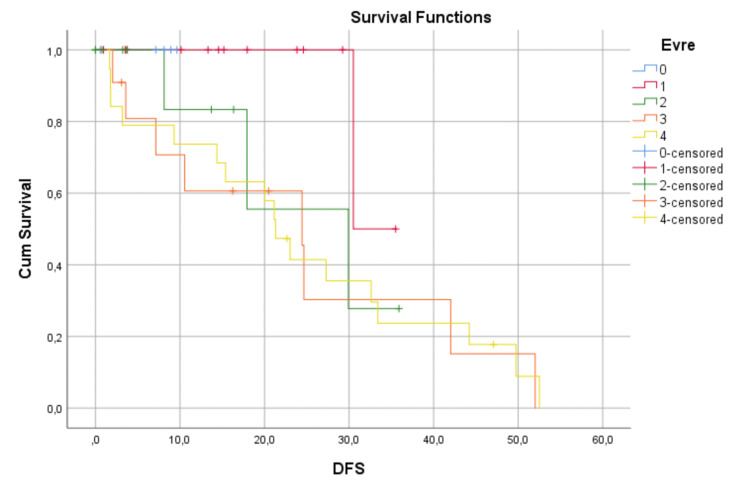
Irrespective of the surgical technique, Kaplan-Meier plot showing DFS DFS: disease-free survival

**Figure 4:**
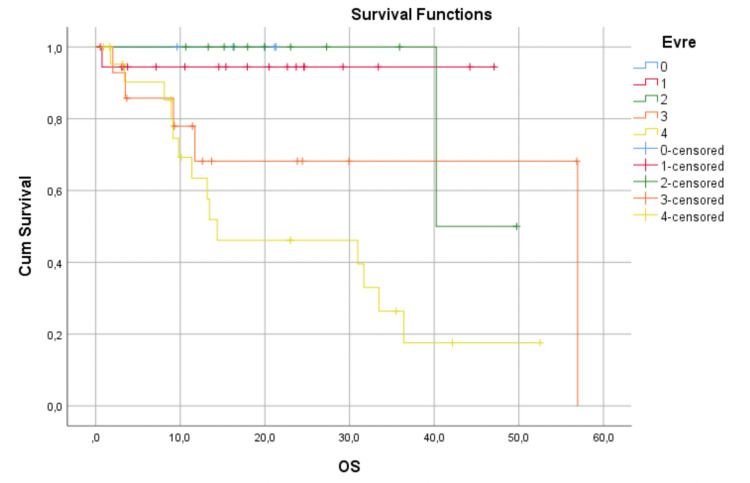
Irrespective of the surgical technique, Kaplan-Meier plot showing OS OS: overall survival

Irrespective of the surgical technique, the median follow-up was 20.7 (range, 2 to 61) months. Also, irrespective of the surgical technique, we can see survival data in Figures 3 and 4.

The OS and DFS times and rates according to the disease stages are shown in Table 5.

**Table 5:** Survival analysis according to disease stages Data are given in median (min-max) or number and percentage unless otherwise stated. MIS: minimally invasive surgery; OG: open gastrectomy; TNM: tumor, node, metastasis, DFS: disease-free survival; OS: overall survival. *Stage 0 included: 3 patient intramucosal tumor, 3 patients regressed than advanced stage tumor to stage 0 after neoadjuvant chemotherapy.

Disease stage	Median follow-up (months)	DFS (%)	OS (%)
0^*^	14.4	100	100
1	17.7	92.3	94.7
2	26.6	72.7	90.9
3	22.9	27.3	64.3
4	20.1	10.5	39.1

## Discussion

In recent years, laparoscopic and robotic surgery have become increasingly used in the treatment of gastric cancer with acceptable early morbidity and mortality rates and favorable oncological outcomes [17]. Nozoe et al. investigated the effect of operating time on the patient outcomes with gastric carcinoma and reported that the operation time was longer in the patients treated with MIS than OG [18]. Similarly, Quijano et al. reported a median operating time of 250 (range, 200 to 490) min in patients undergoing full robotic gastrectomy [19]. Consistent with the literature, the median duration of operation was significantly longer in the MIS group, compared to the OG group (220.45 min vs. 171.06 min, respectively) in our study. The total gastrectomy and subtotal gastrectomy rate was 84.09% and 15.9% in the MIS group, while these rates were 83.8% and 16.1%, respectively, in the OG group, indicating a statistically significantly longer operation time in the MIS group (p<0.0001). Recent studies have demonstrated that MIS is associated with less intraoperative bleeding, postoperative complication, pain, wound infection, and shorter hospitalization than OG [20-23]. In a study comparing the safety and usefulness of laparoscopic total gastrectomy with open total gastrectomy, Sakuramoto et al. reported a mean blood loss of 134±98 mL in the laparoscopic group and 407±270 mL in the open surgery group, indicating a statistically significant difference (p<0.001) [21]. In another study comparing the invasiveness of laparoscopic total gastrectomy and open total gastrectomy, Kawamura et al. reported a mean blood loss of 54.9±45.3 mL in the laparoscopic surgery group and 304.3±237.3 mL in the open surgery group, indicating a statistically significant difference (p=0.000) [22]. In our study, the median amount of blood loss was 142.5 (range, 110 to 180) mL in the MIS group and 180.4 (range, 145 to 230) mL in the OG group (p<0.706), consistent with the literature. Based on the surgical oncology principles, lymph node dissection is the main determinant of disease staging and survival. However, technical difficulties with MIS for adequate lymph node dissection pose a challenge for inexperienced surgeons performing gastrectomy with MIS [23]. Therefore, it is of most importance to perform MIS for gastric cancer in experienced centers by experienced surgeons [11,24-26]. In a case-control study, Caruso et al. compared robotic gastric resection and OG and found no significant difference in the mean number of harvested lymph nodes between the groups (28±11.2 vs. 31.7±15.6, respectively) [27]. In the current present study, the median number of lymph node dissection was 38.9 (range, 15 to 66) and 38.7 (range, 12 to 70) in the MIS and OG groups, respectively (p<0.736), consistent with previous studies using robotic, laparoscopic, and OG [28,29].

In addition, the major complication rate varies between 5.2% and 15.9% for laparoscopic and robotic gastrectomy [30-34]. In the present study, the postoperative Clavien-Dindo grade ≥3 complications were observed in 20.37% of the MIS. This rate was reported as 2.5% in the Japanese cohort [35].

Additionally, the length of hospitalization was shorter in the patients treated with MIS gastrectomy than in OG. In previous studies, the length of hospitalization was reported as 8 to 12.5 days [36-38]. Consistent with these findings, in our study, the length of hospital stay was nine days in the MIS group and 12 days in the OG group. In the current study, the long-term mortality rate was 13.6% (n=6) in the MIS group and 45.16% (n=14) in the OG group, indicating a significant difference between the groups. Among deceased patients, the rate of local recurrence or peritoneal metastasis was 6.81% (n=3) and 38.7% (n=12) in the MIS and OG groups, respectively. The remaining patients died from comorbidities. The rate of recurrence and late mortality was higher in the OG group than in the MIS group (p<0.202). The higher recurrence rate in the OG group can be attributed to the fact that most of these patients were at an advanced stage. Previous studies reported the rate of local recurrence and peritoneal metastasis as 9.76% and 8.42%, respectively [38,39].

Nonetheless, there are some limitations to this study. The single-center, retrospective design with a relatively small sample size and short follow-up are the main limitations. Nevertheless, the conclusion of the article fits within the existing literature. Further large-scale, prospective studies with long-term follow-up are warranted to better understand the use of MIS in daily practice.

## Conclusions

In conclusion, we investigated and compared the outcomes of the minimally invasive versus open gastrectomy techniques in terms of perioperative, postoperative. Despite its technical challenges, MIS seems to be an effective and safe method in treating gastric cancer with favorable early mortality and morbidity rates and long-term oncological outcomes, and acceptable recurrence rates. These conclusions are consistent with the existing literature. This technique can be improved in advanced cancers in experienced centers.
